# API Utilization and Monetization in Finnish Industries

**DOI:** 10.1007/978-3-030-58858-8_3

**Published:** 2020-08-18

**Authors:** Saeid Heshmatisafa, Marko Seppänen

**Affiliations:** 6 IT University of Copenhagen, Copenhagen, Denmark; 7 University of British Columbia, Vancouver, BC Canada; Unit of Information and Knowledge Management, Tampere University, Kanslerinrinne 1, 33014 Tampere, Finland

**Keywords:** API, API economy, Web service, Explorative study

## Abstract

Many companies have followed the trend toward exposing their business assets through open (i.e., Web) application programming interfaces (APIs). However, these firms appear to have adopted API technology largely to meet their customers’ needs and demands. The pressures on industries to develop, implement, and maintain API products and services can prevent companies from gaining a greater awareness of API development’s benefits. Firms may thus miss out on related monetary or non-monetary exploitation of their business assets.

This study explored the status of the API economy and development among Finnish industries. The dataset comprised publicly available information from 226 private and public organizations representing a variety of industries, such as industrial, consumer goods, and services sectors. The current status of API readiness, types, protocols, and monetization models is presented to provide a more comprehensive overview.

## Introduction

In recent years, many companies have started to take advantage of the application programming interface (API) economy. Web APIs have caused disruption because firms can now operate, promote innovation, and create additional value from their business assets with much lower overhead costs [1, 2]. The number of publicly available APIs has thus grown significantly. For instance, ProgrammableWeb reports that over 22,000 APIs are registered on its platform and, on average, 220 new APIs are added every month [3].

Concurrently, developers perceive API as an enabler of software architecture flexibility, efficiency, and agility [4], while API providers seek to seize this as an opportunity to transform their existing business models [5]. For example, Salesforce, a pioneer in customer relations management, offers APIs to increase companies’ system capabilities and integration into their customers’ systems. This transformation of Salesforce’s business model has empowered the firm to handle approximately 60% of its customers’ transactions or about 1.3 billion daily calls through APIs instead of traditional graphical user interfaces. The strategy has generated a new revenue stream of more than five billion United States dollars annually. Another example is the Amadeus IT Group, which operates a travel technology business and generates more than six billion euros in revenue mostly from different API-based solutions.

More recently, API has become a means of developing new business strategies [6]. For instance, eBay’s APIs allow third party users to list auctions and bid, which is responsible for 60% of this company’s annual revenue. In addition, significant proportions of other firms’ revenue, such as 90% of Expedia and 100% of Amazon Web Services, are being generated through APIs [7]. One reason for the API paradigm’s success is its ability to expand ecosystems and increase innovation by embracing the outside-in practice of open innovation. These features allow third-party consumers to create new products or services from one API or a combination of available APIs, thereby enabling new approaches to capturing and creating value [8]. APIs are, therefore, becoming the corporate world’s central focus.

This paper presents the initial results of a study that explored the current status of the API economy in various industries and sectors. Previous research has focused on large multi-national companies, so scant attention has been paid to small and medium-sized enterprises (SMEs) in this context. The API economy’s development within SMEs was thus included in the present study. An explorative analysis was also conducted to assess the popularity of this phenomenon by addressing the following questions:

- How many organizations have adopted APIs?
- What are the most common API types and protocols?
- What monetization models are used?

## Background

The buzzword “API economy” has recently started to attract much attention among scholars and practitioners, but the concept of API is not new. This term was initially coined in 1968 with reference to a framework or library for a specific programming language [9]. In the 2000s, the advent of service-oriented architecture (SOA) created an opportunity for companies to build business-to-business relationships using standard interfaces via a simple object access protocol (SOAP) [7]. The concept of SOA was then developed further by employing Web-based technologies such as representational state transfer (REST) [10].

With the evolution of REST, Web API shifted developers’ approach to constructing and publishing applications over the Internet and created the culture of reusability by adapting create, read, update, and delete interfaces. Currently, software as a service, platform as a service, and infrastructure as a service enable developers to publish and manage application composites using key access. Developers have moved into a new era focused on speed rate, innovation, and performance while becoming better at controlling costs and risks [11].

In general, API is a way for two applications to communicate with each other over a network using a common bilateral language [6]. Thus, API acts as a control point at which a compilation of services is exposed to potential users in a controlled, managed manner [11]. In the context of the API economy, API, Web API, and business API are used interchangeably, functioning as a meta concept. Obtaining economic benefits from API is often referred to as the “API economy,” which is defined as “an economy in which companies expose their (internal) business assets or services in the form of (web) APIs to third parties with the goal of unlocking additional business value” [12]. Consequently, a more appropriate definition of Web API is an Internet-based software interface that publishes specific business assets in a controlled manner [12].

Typically, this new economic model consists of three key players: API providers, API consumers, and end-users [2]. Providers expose their business assets (i.e., products, services, or/and data) over an API, and consumers are the businesses that take advantage of one API or a combination of APIs to develop new products, services, or results. End-users, in turn, are users who have a direct relationship with consumers.

Diverse and yet compatible API business models have been created [13], including free, paid, and indirect models. In paid business models, the developers either pay or get paid for API usage, while, in indirect business models, consumers are subject to business models such as content acquisition and content syndication. In this context, consumers can become the providers’ direct customers by using the exposed utility services. In addition, providers may sell core products through an API, while consumers play the role of resellers [2]. Providers can exploit APIs in a great number of ways, and many of these fit into both categories or in between.

What makes APIs unique is that providers often operate in a black box and expose their business assets without being aware of the business opportunities that APIs offer and the ways that consumers can use APIs to innovate. The potential benefits of the API economy are as follows [1]:

- Reducing development costs and time
- Staying relevant in the market
- Reaching diverse platforms and devices
- Focusing on core values by outsourcing production to API consumers
- Capitalizing on new partnerships
- Entering new customer bases
- Increasing brand loyalty
- Inspiring industry standards and user expectations

Regarding types of APIs, the most common classifications are open data, open and/or public or partner, and internal and/or private APIs [14]. Open data APIs are openly accessible information mostly provided for “free” from organizations such as governments and schools. Open APIs are associated with Web APIs, which are the present study’s focus and which include public and partner APIs. Open public APIs are publicly available as they can be accessed by anyone without establishing a business relationship. Conversely, partner APIs are only accessible with a key after a partnership or customer agreement is signed.

## Research Method

Given this research’s aims, a quantitative descriptive statistics method was chosen to explore the characteristics of the API economy within the sample of organizations. First, the dataset was collected from diverse private and public organizations operating in Finland, resulting in a final dataset on 226 organizations, a list of which is available upon request in a Google sheet format. The first category in the dataset comprises the top 100 companies in Finland based on their turnover in 2019 [15]. The second category includes 126 convenience sample-based SMEs to provide a more comprehensive overview of the topic under study.

Second, four variables—API readiness, types, protocols, and monetization models—were selected to define the dataset’s demographics. Third, secondary data such as publicly available white papers and companies’ official websites were carefully examined. Last, the content analysis’s results were evaluated and associated with the findings for the defined variables. When a company did not clearly mention any of the variables, the keyword “unknown” was used.

The required data were gathered in March 2020, and the results were processed and stored in Microsoft Excel spreadsheets. Next, data wrangling was applied to clean the dataset by removing different errors, nulls, and duplications. The purified dataset was further analyzed by creating pivot tables to represent the variables’ demographics. Finally, the relevant tables were constructed using Tableau software to validate and facilitate a more in-depth examination of the results.

## Results

Information was gathered from 226 companies and organizations from different industries such as industrial, consumer goods, and services sectors. The results show that, out of 226 firms, only one-third (number [n] = 77, 34%) have open APIs, and two-thirds (n = 149, 66%) do not participate in APIs publicly (see Table 1).

**Table 1.** Distribution of APIs across industries.

	With API	Without API	Total
Industrial	18	56	74
Consumer goods	3	31	34
Consumer services	10	23	33
Public	20	1	21
Technology	15	5	20
Financial	5	6	11
Basic materials	1	8	9
Healthcare	1	7	8
Materials	0	6	6
Oil and gas	0	3	3
Utilities	1	2	3
Consumer services	2	0	2
Telecommunications	1	1	2
**Total**	**77**	**149**	**226**

**Fig. 1.**
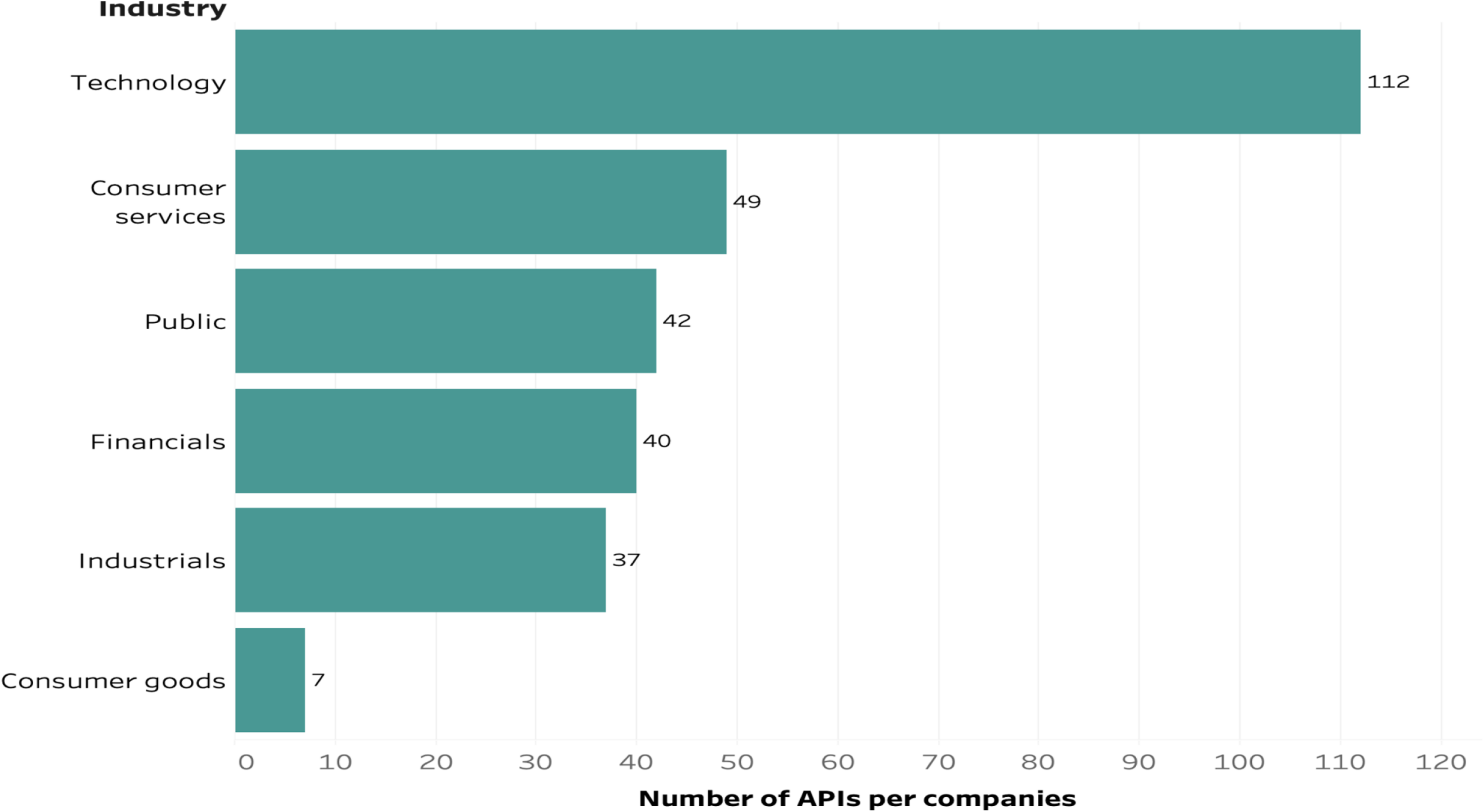
API distribution across selected industries (more than one API).

The distribution of APIs indicates that the public sector is at the top of open API development with 20 organizations, whereas the healthcare sector, with its vast potential and high volume of data, has not yet invested in this technology substantially. However, technology companies have produced the most APIs or approximately 40% (n = 112) of the total. Notably, some organizations have several APIs (see Fig. 1). Consumer services firms come second with a total number of 49 open APIs. In addition, none or only one API was identified in six industries: basic materials, healthcare, telecommunications, utilities, materials, and oil and gas.

Companies apparently tend toward investing in one API type. Firms have also published more open public APIs (n = 38) compared to any other types. The second most common API type is open partner APIs (n = 18). Only a small fraction of the companies under study have APIs in both public and partner formats (n = 8). Thirteen companies did not explicitly state their type of API in any of the documents examined.

Regarding monetization models, the most common revenue model in the sample is “free” with 39 companies, which means that no direct earnings or pricing is linked to API usage. A further 32 cases fall into the category of “unknown,” indicating that a monetization model was not specified. Evidently, companies tend to use APIs as a facilitator and an added value to offer their existing customers.

The matrix in Table 2 shows that the 31 companies with open public APIs are exposing their business assets for free. Only a small number of the organizations in question have any monetization models linked with their APIs. All the API types and monetization models counted, totaling six organizations, are shown in italics in Table 2. Surprisingly, 12 companies did not mention their type of API or revenue model. In total, 32 organizations did not clarify their revenue model and so were coded as “unknown.”

**Table 2.** Matrix of API classifications and monetization models.

	Unknown	Free	Free & Freemium	Free & Freemium & Premium	Free & Premium	Premium	Total
Open API (Public)	7	31	*0*	*0*	*0*	*0*	**38**
Open API (Partner)	11	6	*0*	*0*	*0*	*1*	**18**
Unknown	12	1	*0*	*0*	*0*	*0*	**13**
Open API (Partner & Public)	2	1	*1*	*2*	*1*	*1*	**8**
**Total**	**32**	**39**	***1***	***2***	***1***	***2***	**77**

Overall, the results indicate that companies do not clearly determine a revenue model. In most cases, consumers are mandated to fill out an application or contact the providers directly to receive detailed information about the type of contract and pricing models. In addition, in freemium models, companies expect consumers to upgrade to a premium model after a specific number of calls.

Similar to the findings for revenue models, API customers are also left ambiguous. Most publicly available APIs (n = 45) do not target specific market segments, and companies are unclear about whether the assets are offered to developers and/or their business partners. However, 17 open APIs expose business assets as an added value offered to partners, whereas only 3% (n = 9) of the APIs are used to collaborate with both developers and business partners. Furthermore, six public APIs explicitly aimed to serve developers.

Private companies also devote a portal to provide documentation on their APIs, but this is true of less than one-fifth (18%) of these firms. About half of the companies publish their business assets through third-party platforms such as GitHub, so the results show that only one out of six (16%) private companies dedicate an official portal to providing documentation and examples to consumers.

One-third (33%) of providers further do not clarify where consumers can access the APIs mentioned. Notably, most of the companies’ information was collected for this study from their annual report, blogs, help portal, or even GitHub. This poor documentation of official information on companies’ assets reflects the high level of uncertainty firms experience concerning their business assets.

**Fig. 2.**
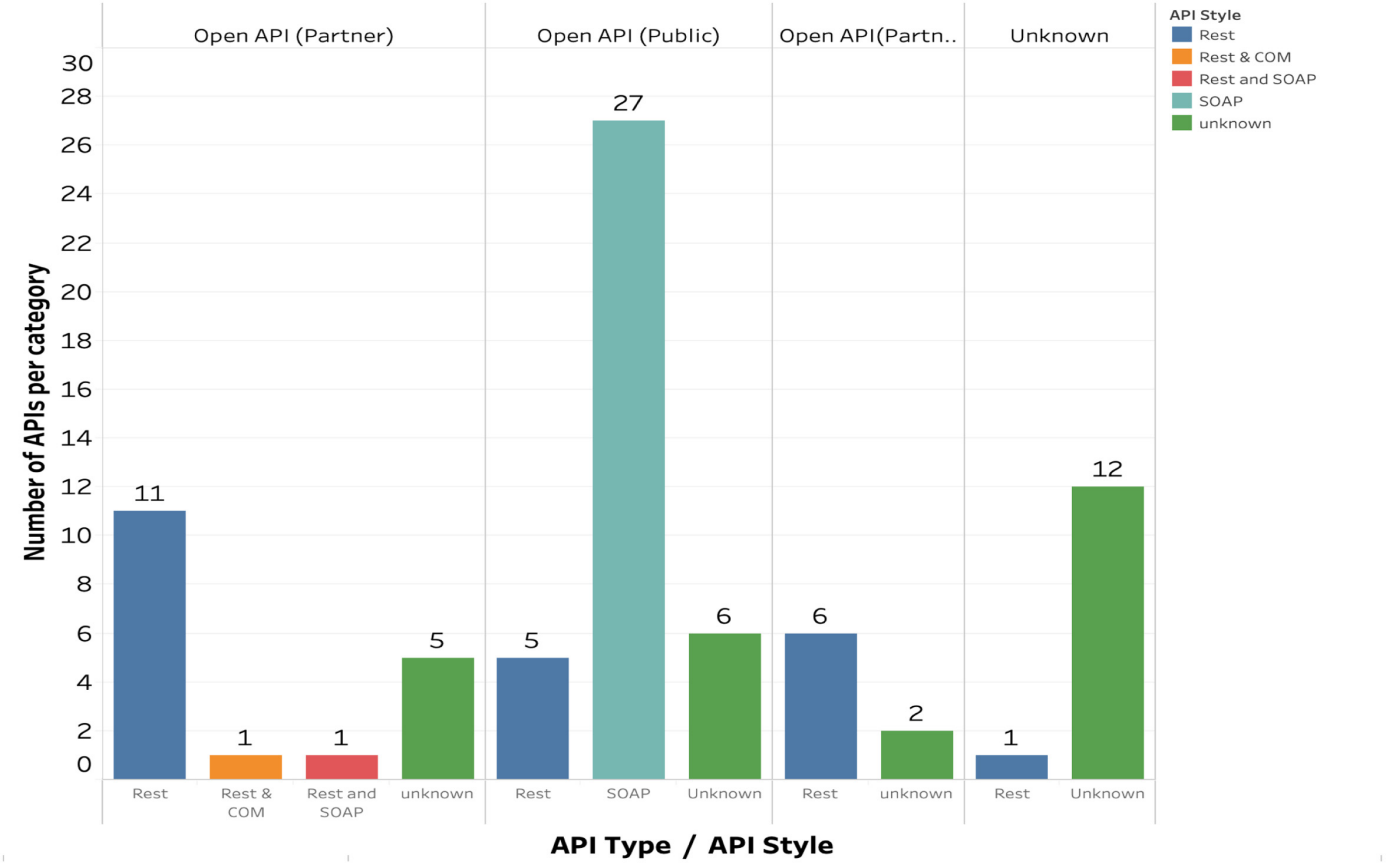
API types versus style.

In addition, SOAP architecture is more prevalent among open public APIs (see Fig. 2), while REST architecture is more common among partners and hybrid APIs. At least two firms with open partner APIs also appear to offer two different API styles (i.e., protocols) to consumers to increase the information’s flexibility and usability.

## Conclusion

This paper provides an overview of public APIs and their monetization models among Finnish industries, based on a broad sample of 226 private and public organizations. Despite the growing trend toward developing open APIs, the results show that only one-third of these firms provide public access to their Web APIs. Industrial companies are pioneers in this area, but, rather surprisingly, even high potential sectors such as healthcare do not show much sign of making APIs generally available.

In terms of monetization, the findings include that the most popular revenue model among the firms under study is still “free,” so direct monetization has not yet been established. Companies appear to provide APIs as an added value to their business partners, as well as to remain relevant in the market. In addition, even though documentation and protocols are some of the effective ways to encourage and engage with consumers, the results of the study indicate that companies have invested rather little on the subject matter.

This study had a few limitations that are worth mentioning. First, given the exploratory nature of the research, the results are only preliminary and are not generalizable to any great extent. Second, the data selection process was based on convenience and accessibility. Last, the results are descriptive and based on publicly available sources. Therefore, the dataset did not allow an assessment of to what extent the organizations in question may use APIs internally. Further studies using case study or survey data could help to shed light on this internal use. In addition, future research may benefit from a more theoretical approach to monetization strategies and causal relationships between monetization and organizations’ performance.
